# Insights on Allergic Rhinitis Management from a Northern Cyprus Perspective and Evaluation of the Impact of Pharmacist-Led Educational Intervention on Patients’ Outcomes

**DOI:** 10.3390/medicina54050083

**Published:** 2018-11-07

**Authors:** Günay Arsoy, Ahmet Varış, Louai M. Saloumi, Abdikarim Abdi, Bilgen Başgut

**Affiliations:** 1 Department of Clinical Pharmacy, Faculty of Pharmacy, Near East University, Nicosia, Northern Cyprus, Mersin 10, Turkey; louaisaloumi@hotmail.com (L.M.S.); daud87@hotmail.com (A.A.); bilgenbasgut@gmail.com (B.B.); 2 Department of Otolaryngology, Burhan Nalbantoglu State Hospital, Nicosia, Northern Cyprus, Mersin 10, Turkey; ahmetvaris@mynet.com

**Keywords:** allergic rhinitis, Northern Cyprus, ARIA, ENT specialist, community pharmacist

## Abstract

*Background and objective*: the global prevalence of allergic rhinitis (AR) is rising and yet there is scarce information concerning the diagnosis, management and treatment patterns of AR in Northern Cyprus (NC). This study aims to provide a unique perspective on AR management as well as assessing the effectiveness of the pharmacist-led educational intervention for improving care of AR patients. *Methods*: across-sectional survey was carried out with community pharmacists (*n* = 70), patients (*n* = 138) and ear, nose and throat (ENT) specialists (*n* = 12) in NC. For a controlled interventional trial, trained pharmacists provided a brief education on management of AR and nasal spray technique for patients while other pharmacists provided the usual care. Quality of life (QoL) and other outcome measures on the perceived symptom severity of the two groups were compared after a 6-week period. *Results*: only 33.3% of the ear, nose and throat (ENT) specialists and 15.7% of the community pharmacists are aware of the Allergic Rhinitis and its Impact on Asthma (ARIA) guidelines. The majority of patients (63%) self-managed with over-the-counter medications. Nasal congestion (96.4%) is the most bothersome symptom and oral antihistamines are the most commonly purchased medications (51.4%), indicating a pattern of suboptimal management. The pharmacists-led educational intervention has resulted in statistically more significant improvement in regards to nasal congestion and QoL for the intervention group patients (*p* < 0.05). *Conclusion*: the current management of AR has not been in accordance with the ARIA guidelines in NC. An educational intervention of the pharmacists can enhance the symptom management and improve the QoL in patients with AR.

## 1. Introduction

Allergic rhinitis (AR) is an immunoglobulin E (IgE)−mediated inflammatory reaction in the nasal mucosa caused by inhaled allergens, such as pollen, mold or animal dander [1]. The high prevalence of AR and its effect on quality of life (QoL) have led to its classification as a major chronic respiratory disease [2]. AR affects 10% to 40% of the global population, rates that are still increasing [3]. In Northern Cyprus, it is estimated that 40% of the population suffer from AR, which is nearly half of the entire population. AR can significantly reduce QoL [4], impair sleep and adversely affect leisure activities, social life, school performance and work productivity [5,6]. Cardinal symptoms of AR are rhinorrhea, nasal congestion, nasal itching and sneezing, all of which are spontaneously reversible or controlled by adequate treatment [7,8].

AR is frequently associated with other co-morbidities including rhinosinusitis which is present in 20–30% patients [9], asthma (10–40%) [10,11], otitis media with effusion, conjunctivitis and nasal polyps [12]. This aspect makes the diagnostic approach sometimes particularly complex [13]. Many patients who suffer from AR do not recognize the process as such. Since AR itself is not life-threatening, it is considered by many to be a trivial disease that can be managed by the patient himself or by healthcare providers other than physicians [14]. In this context, community pharmacists are usually the first line of contact for AR patients [7,15], also because, several medications are available as over-the-counter (OTC) [16].

As the most easily accessible and trusted healthcare professionals (HCPs), it is important for community pharmacists to be able to accurately recognize AR and assess its severity. If management in pharmacy is appropriate, they should provide patient counseling while dispensing OTC drugs and, in severe cases, they should refer patients to healthcare specialists for further treatment [17]. Therefore, community pharmacists represent an important healthcare resource in mediating the contact between patients and physicians [14], in establishing the optimal therapy, and in managing good disease control.

The Allergic Rhinitis and its Impact on Asthma (ARIA) guidelines are the recognized worldwide authority on AR diagnosis and management [15,18]. These guidelines classify AR according to its duration and severity, provide an evidence-based recommendation for available treatments, and propose a step-wise approach for managing AR [13]. The primary purpose of these guidelines is to address quality improvement opportunities for all clinicians, in any setting, who are likely to manage patients with AR, as well as to promote effective diagnosis and therapy [19]. So, awareness of ARIA guidelines by HCPs and managing AR in accordance with guideline recommendations is really important for patient outcomes.

Patient education is considered a major intervention for managing allergic rhinitis alongside environmental control, pharmacotherapy and allergen immunotherapy according to ARIA guideline recommendations. Education may involve, essentially, the role of both allergic and non-allergic triggers, patient illness perception and concerns regarding medicine and counseling on nasal spray administration techniques [20]. HCPs-led educational interventions were reported to improve the QoL in AR patients and enhance their self-management [17].

The aim of this study is to fill gaps in our knowledge on the current diagnosis, treatment and management of AR in Northern Cyprus and to assess their compliance with the ARIA guidelines by surveying community pharmacists, AR patients and their ear, nose and throat (ENT) specialists. This study also aims to evaluate the effectiveness of the pharmacist-led educational intervention for improving patients’ QoL and perceived symptom severity.

## 2. Materials and Methods

### 2.1. Study Design and Participants

**Figure 1:**
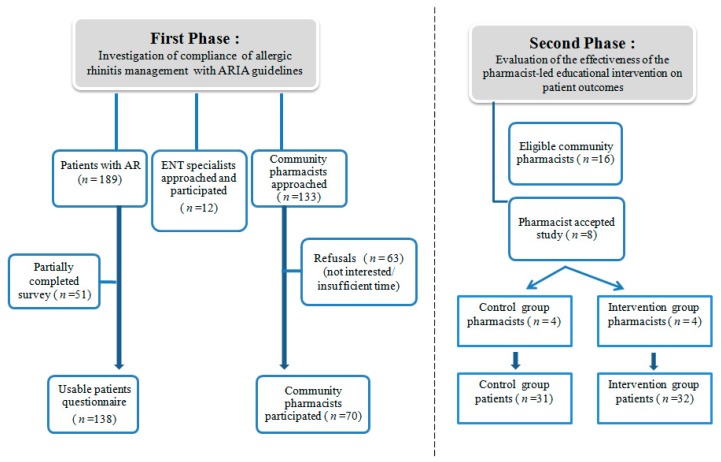
Inclusion of participants in the study.

The study involved two phases. The first phase investigated the AR management of the ENT specialists, community pharmacists and AR patients. The second phase evaluated the effectiveness of the pharmacist-led educational intervention on patient outcomes (Figure 1).

In the first phase a cross-section of all participants, were separately assessed using questionnaire-based surveys. A list of 218 licensed community pharmacists was obtained from the Cyprus Turkish Pharmacists Association. Our study sample included only experienced community pharmacists with 5 or more years’ experience as stipulated by Turkish and Northern Cyprus Pharmacists’ Association’s norms. So, 133 of community pharmacists were contacted and invited to participate in the study. AR patients who were aged over 18 and presenting a prescription that included AR medication in their own name were invited to participate in the study. In Northern Cyprus, there are 31 ENT specialists registered with the Cyprus Turkish Medical Association and only 19 of those have been carrying out their occupation. In our study, a purposive sampling method was used and all ENT specialists who had diagnosed those patients included in the study were contacted and invited to participate (*n* = 12; in private practice or hospital practice throughout Northern Cyprus); all of them agreed participation.

The second phase was a randomized controlled trial (RCT), conducted with the sub-sample of community pharmacists. Sixteen pharmacists who had participated in the first phase and had reported to have more than 100 patients diagnosed with AR per year were invited to participate in this study; 8 of them agreed to collaborate with the research team. These pharmacists were randomly allocated into two groups, either the intervention group (*n* = 4) or the control group (*n* = 4). Pharmacists who were randomized to the intervention group attended an intensive, evidence-based 1-day educational training on the ARIA Pocket Guide for Pharmacists delivered by the research team [21]. Each pharmacist was requested to recruit patients to participate in the study. Patient participant inclusion criteria for the study were: presenting prescriptions that included a nasal spray for AR; over the age of 18; and able to revisit the pharmacy after 6 weeks. Participants were excluded if they were: presenting prescriptions for other patients, refusing to participate, pregnant or breastfeeding and/or terminally ill.

### 2.2. Research Instruments

#### 2.2.1. Development of the Questionnaires

Previous questionnaires used in identifying ENT specialists or physicians, pharmacists and patients perspectives in management and treatment of AR were reviewed [14,22,23] and questionnaire items were developed accordingly by a panel of experts. The panel of experts comprised of a pharmacologist, a clinical pharmacist, a community pharmacist and two ENT specialists practicing in Northern Cyprus. The panel helped to develop and validate a modified version of the questionnaires for each of the ENT specialists and pharmacists with slight modifications taking into account the role played by each group in the healthcare system.

The final structured questionnaires consisted of 11 items for ENT specialists, 12 for community pharmacists and 16 for AR patients (all of them with closed- and open-ended questions). All questionnaires could be completed within 10 min. The draft questionnaires were distributed to four randomly selected community pharmacies and an ENT specialist to assess its readability and content validity. They were also pretested among a group of patients at a community pharmacy for clarity, relevance, acceptability, and time to completion. Participants of the pilot study and expert panel were not included in the study sample.

The ENT specialists’ questionnaire records the following information: physician demographics, length of practice, the number of AR patients seen per month, seasonal incidence of AR, practice in relation to diagnosis of AR (including diagnostic tools and tests), symptoms considered when diagnosing AR, practice relating to the treatment of AR patients (including most frequently prescribed treatments and modalities), reports related with compliance and adverse effects, patient education and awareness of the ARIA guidelines.

The pharmacists’ questionnaire records similar information to that described above for ENT specialists. Pharmacists were also questioned on management plans considered for special patient populations and medications most commonly prescribed by ENT specialists. The question which related with tools and tests used for diagnosis of AR were not included in pharmacists’ survey.

The patients’ questionnaire records the following information: patient profile (age, gender, education level, location, contact details and ENT specialist’s contact details), bothersome symptoms that prompted them to visit physician, onset and severity of symptoms (a visual analogue scale was used for assessing severity), treatments used and satisfaction with treatment, immunotherapy experience, role of pharmacists and ENT specialists in patient’s diagnosis and treatment, impact of AR on patient’s QoL, perceived allergens/causes and sources of information about AR.

#### 2.2.2. Assessment Tools for Intervention Study

The impact of pharmacist-led intervention was assessed using two validated assessment tools: a Mini Rhinoconjunctivitis Quality of Life Questionnaire (Mini RQLQ©) and a visual analogue scale (VAS) [24,25]. The Mini RQLQ has 14 questions in five domains (activity limitation, practical problems, nose symptoms, eye symptoms, and other symptoms) that answered on a 6-point Likert scale [25]. The VAS is a 10-point scale (where 10 = as bad as symptoms could possibly be and 0 = no symptoms) that is used to measure perceived symptom severity.

### 2.3. Data Collection

The ENT specialist and pharmacist surveys were completed by face-to-face interviews between March and May 2017. AR patients were recruited from community pharmacies that participated in the study. The eligible patients were asked by the pharmacists to answer the self-administered questionnaire. If they agreed, they answered it within the pharmacy.

The second phase of the study involved two visits: baseline (V1) and 6 weeks post baseline (V2) for each patient. All patients completed the questionnaire that was developed for the first phase of the study at V1. In addition, they scored the Mini RQLQ and VAS at each of their visits. For intervention group patients, V1 consisted of a brief intervention by trained pharmacists. The intervention focused on AR education, counseling on identification and avoidance of AR triggers, and teaching of nasal spray technique. Those patients were also provided with a brochure on the identification and management of AR. The control group patients received usual care and standard communication regarding their medication. Any inquiries addressed to the pharmacists were answered according to routine practice in the pharmacy.

### 2.4. Ethical Considerations

Ethical approval for this study was obtained on 17 November 2016, from the Institutional Review Board (IRB) of Near East University Hospital (YDU/2016/41-337). Patient consent forms were obtained and research was conducted in accordance with the Declaration of Helsinki.

### 2.5. Statistical Analysis

Data analysis was performed using SPSS Statistics (Statistical package for social sciences, IBM, USA, version 21). Participant characteristics (age, gender, experience) were expressed as mean ± standard deviation (S.D.) and frequency. The Kolmogorov–Smirnov test was carried to verify data distribution. Due to the non-normal distribution of data, group comparisons (AR-related symptom severity and QoL) were made using the non-parametric Wilcoxon signed-rank test for within group differences, and the Mann–Whitney U test to compare the two groups at baseline as well as the respective groups’ change scores over time. These data were expressed as median and interquartile range.Statistically significant differences between 2 variables were accepted when the probability of significance (*p* value) was < 0.05.

## 3. Results

### 3.1. Response Rate and Participant Characteristics

Of the 133 pharmacists contacted, 70 pharmacists agreed and participated in the first phase of the study (response rate of 53%). All ENT specialist (*n* = 12) invited agreed to participate in the study.

A total of 189 patients with AR were recruited to the study. Of them 51 patient surveys were excluded from the study because of being only partially filled. A total of 69 patients agreed to participate in the second phase of the study; 6 patients did not return for V2 and their data was excluded. At the end of the study 32 participants from the intervention group and 31 from the control group completed V2, representing 94% and 89% completion rates of enrolls, respectively.

ENT specialists were predominantly male (75%). The mean age was 45.75 (±10.35) years and the average year of experience was 17.08 (±10.57) years. The mean age for the pharmacists was 38.56 (±13.45) years; 70% were female and the average year of experience was 14.27 (±11.82) years. The mean age for the patients was 34.33 (±13.28) years and 54% were female. The education level of patients in this study was quite high, 49% had graduated from high school and 44% from university.

The mean age of intervention group participants was 32.7 (±11.9) years and 53.1% were male. The mean age of control group participants was 31.1 (±13.8) years and 54.8% were female; 50% of intervention group patients and 48% of control group had graduated from university.

### 3.2. First Phase Allergic Rhinitis (AR) Experiences and Management Questionnaire Responses

#### 3.2.1. Data on Patients’ Experiences

According to the results, symptoms of AR were mostly experienced in spring (49.3%), followed by autumn (39.9%). Some patients (20.3% of sample) experience AR symptoms throughout the entire year. There was a wide time span between the onset of symptoms and diagnosis of AR by physicians. Only 15.2% of patients visited or were referred to physicians within 5 days following the onset of symptoms, while 46.4% visited a physician more than 30 days after the onset of their symptoms. A majority of patients (58.4%) reported that they decided to visit a physician themselves. Of the others, 24.8% stated that they were referred by pharmacists, 8.8% by other specialists and 8% by friends/family members.

The most frequent complaint prompting patients to visit ENT specialists was nasal congestion (96.4%) (Table 1). Analysis of VAS showed that 91.2% of patients suffered from nasal congestion and had moderate-to-severe AR. Also 63% of patients with undiagnosed AR resorted to self-treatment with OTC medications. Of these 81.6% used OTC medications on the recommendation of a pharmacist, and the remaining patients, either self-selected (17.2%) or consulted friends/family members (1.14%). Nearly half of the patients (42.8%) reported that an intranasal corticosteroid (mometasone furoate) produced significantly higher treatment satisfaction than other OTC medications used for AR treatment. The most commonly purchased products were oral antihistamines (51.4%) and cetrizine (54.9%). The most common drug-related problem (DRP) reported by patients (64.3%, *n* = 42) was the adverse effects of first generation antihistamines including drowsiness and sleepiness. Nosebleeds, the most common limiting side effect of intranasal corticosteroids (INCs) was also experienced by patients (35.7%, *n* = 42). Only 3.6% of patients experienced allergen-specific subcutaneous immunotherapy for AR. Regarding medication use 66.7% of patients reported that they use medication only as needed. When the symptoms reoccur, 58.7% take the same medicines previously prescribed by their physician, 35.5% contact with the physician and 5.8% consult their pharmacists. Patients’ answers showed that seasonal changes (45.7%) were the most common trigger identified (Table 1). When patients were asked how AR affects their QoL, 57.2% said AR causes troublesome symptoms (Table 1). Most patients (93.5%) stated that they were informed by their physician. Of these, 54.3% were also informed by their pharmacists.

#### 3.2.2. Data on Pharmacists’ Practices

Regarding pharmacists’ responses to questionnaire, 37.1% reported that between 30 to 100 patients diagnosed with AR visit their pharmacy every year. AR patients were most commonly seen in the spring and autumn (77.1% and 64.3%, respectively).

Nasal secretion (42.9%) was considered by pharmacists as the chief symptom that made them suspect AR (Table 1). The majority (62.9%) of pharmacists preferred the management pathway of starting treatment and then referring them to ENT specialists; only 10% follow-up patients after starting symptomatic treatment. Pharmacists reported recommending oral antihistamines very frequently for AR treatment (Table 2). About 80% of pharmacists reported that they have never recommended leukotriene receptor antagonists (LTRAs), anticholinergics and oral corticosteroids (Table 2). When asked which products are prescribed by ENT specialists most frequently for AR, pharmacists mentioned branded cetrizine (37.1%) most frequently, followed by mometasone (31.4%) and desloratadine (27.1%).

Pregnancy (94.3%), breastfeeding (88.6%) children under the age of 12 (88.6%), and symptoms of asthma (75.7%) were the main circumstances in which referral to ENT specialists is preferred by pharmacists. When pharmacists were asked whether they received complaints about side effects of allergy medications, 54.3% said yes. Sedation, dryness of nasal mucosa and nosebleed were the most commonly experienced side effects reported by pharmacists. Only 27.1% of pharmacists reported compliance-related problems in which the great majority of patients (79%) stop taking a prescribed medication without the advice of their physician or use medication more frequently than prescribed. Majority of pharmacists (73%) reported educating their patients about allergen avoidance.

#### 3.2.3. Data on Ear, Nose and Throat (ENT) Specialist’s Practice

Half of the ENT specialists reported seeing an average of 15 AR patients per month. The majority of ENT specialists (75%) said that AR patients generally experience symptoms in the spring, followed by autumn (50%). About 67% of ENT specialists reported that patients prefer to visit them on their own initiative. Only 8.3% reported referral by pharmacists. When making the diagnosis of AR, ENT specialists said they based treatment on a patient’s history and anterior rhinoscopy (66.7% and 33.3% respectively). They reported that the symptom most frequently seen in the AR patients who consulted them was nasal congestion (58.3%) (Table 1). Of ENT specialists, 75% reported that intranasal corticosteroids (INCs) were the most frequently prescribed products for AR patients, followed by oral antihistamines (66.7%) (Table 2). Some of the ENT specialists reported that they had never prescribed anti-cholinergics and LTRAs (41.7% and 33.3%, respectively) (Table 2). Antihistamine alone was the first choice of treatment for all symptoms of AR, except nasal congestion. For the treatment of nasal congestion, 41.7% of ENT specialists prescribed INCs while antihistamine alone was prescribed by none of them. Combinations of antihistamines and INCs were preferred in the treatment of nasal secretion (25%) and nasal itching (41.6%). Poor patient adherence to long-term treatment was reported by half of the ENT specialists. About 50% of ENT specialists received complaints about the adverse effects of medications. Sedation, dryness of the nasal mucosa and nosebleeds were the most commonly reported adverse effects. All of the ENT specialists reported that they informed their patients about diagnosis, treatment and management of AR.

### 3.3. Second Phase Controlled Interventional Study Data

#### 3.3.1. Patient Rated Symptom Severity

At baseline (V1) there was no statistical difference between AR symptom severity scores in intervention and control group patients (*p* > 0.05). A comparison of perceived symptom severity scores between V1 and V2 showed that for both groups there was a significant decrease in symptom severity scores over the study period (*p* < 0.05). There was also a significant difference between intervention and control group at V2 regarding the nasal congestion severity score (*p* < 0.05) (Table 3).

#### 3.3.2. Quality of Life Measurements

At baseline (V1) there was no difference in the Mini RQLQ© Subscale scores between intervention and control group participants (*p* > 0.05). Comparison of the subscale scores between V1 and V2 revealed that all subscale scores were significantly decreased over the study period. However, a different pattern of improvements was observed between two groups with the exception of the eye symptoms subscale at V1/V2 (*p* > 0.05) (Table 4).

## 4. Discussion

This was the first study that assessed the current diagnosis and treatment patterns related to the AR management of ENT specialists and community pharmacists, based on their clinical experience and patient interaction, from a Northern Cyprus perspective. It also gives a significant insight into patients’ behaviors and attitudes to this prevalent condition and its treatment.

In addition, for the first time, an intervention study was designed to evaluate the effectiveness of the pharmacist-led education on AR patients’ outcomes. This study provided some feedback on delivery and acceptability of the intervention as well as suggesting that medications alone are not always the most effective way to improve symptom severity and ultimately patients’ QoL. The data showed the need for better patient education and guideline-directed management by pharmacists to improve disease control.

AR is on the increase and becoming ever more challenging to treat so it is more important than ever that HCPs have the most up-to-date evidence on the best treatments [23,26]. The ARIA guidelines set out evidence-based standards of best practice for the diagnosis and treatment of AR. There are specific ARIA guidelines for pharmacists detailing all aspects of AR diagnosis and treatment, with advice given on when referral to a physician is appropriate [21]. However, this study suggested that only 33.3% of ENT specialists in Northern Cyprus are aware of these guidelines, despite seeing an average of 15 AR patients a month. Awareness is even lower among community pharmacists, at just 15.7%, even though the vast majority of participating pharmacists estimated that at least 30 AR patients visit their pharmacy per year. Similarly, in a survey study performed in Italy with 100 pharmacists, Canonica et al. (2015) revealed that only 13% of pharmacists were aware of ARIA guidelines [22]. In another study done in Asia, Recto et al. (2017) revealed a high awareness of the ARIA guidelines among allergists, dermatologists and ENT specialists. Eighty percent of ENT specialists generally follow ARIA guidelines for AR treatment [27]. Prepageranet al.’s survey done in Malaysia showed high satisfaction with the recommendations from the current ARIA guidelines; between 58% and 89% of ENT specialists, pharmacists, and GPs [28]. Clearly, education programs need to be undertaken to increase awareness of and adherence to ARIA guidelines.

According to ARIA classifications, the patients who have one or more symptoms of; impairment of sleep, daily activity or work in school or troublesome symptoms have moderate to severe disease and those who do not have these problems have mild disease [21]. This indicates that all patients included in this study had moderate-to-severe AR. Nonetheless, a vast majority of them (63%) considered AR as a trivial disease that can be managed by the patient himself, and the pharmacist, therefore, represented the firstline of contact. In this instance, for the pharmacist it is important to be able to accurately recognize AR, assess its severity and make a guideline-informed treatment recommendation, whether it is medication or referral to a physician. Tan et al. conducted a survey study of 296 pharmacy customers purchasing treatments for nasal symptom(s) from community pharmacies and found that a majority of participants (69%) that self-selected OTC medications experiencing AR symptoms (39% persistent and 30% intermittent AR) and only 44.3% of the subgroup had a doctor’s diagnosis [29]. In the same study, 25 participants required immediate referral to the doctor. This highlights the importance of pharmacist’s engagement with patients, even for what are perceived to be trivial symptoms.

To aid the implementation of a stepwise approach to patient management, ARIA introduced a patient classification based on the AR symptoms’ time patterns (“intermittent” vs. “persistent”) and severity (“mild” vs. “moderate/severe”) [24]. According to ARIA guidelines, AR that is intermittent or mild may be appropriate for management by the pharmacist. Referral to a physician should be considered in the case of symptoms that are moderate to severe-persistent and for symptoms poorly responsive to treatment after 2 to 4 weeks [7]. However, only 10% of pharmacists followed-up their patients after starting treatment. A vast majority of them (63%) preferred the management pathway of starting treatment and then discharging patients to a physician for follow-up. In a survey study in Malaysia, Prepageranet al. found that pharmacists preferred to review their patients for mild AR and moderate-to-severe AR after 2–4 weeks of initial therapy that is in line with the ARIA guidelines (11% and 36%, respectively) [28].

Antihistamines and LTRAs are recommended as the firstline for patients with mild symptoms, while INSs are considered the firstline for patients with moderate-to-severe AR [18,30]. This survey revealed a significant mismatch between treatment patterns among ENT specialists and pharmacists and the ARIA recommendations. Eighty percent of pharmacists and 50% of ENT specialists reported that they have never advised LTRA for their patients. Most of the patients presenting to ENT specialists had moderate-to-severe AR, yet the most frequently prescribed AR treatment was an antihistamine alone, which is not concordant with the ARIA guidelines. Seventy five percent of ENT specialists reported that they prescribe INCs very frequently. The results of symptom-based treatment patterns of ENT specialists showed that INCs were preferred over oral antihistamine only for the treatment of nasal congestion. Although, moderate-to-severe AR that is not persistent may be appropriate for management by pharmacists, survey results showed that 60% of pharmacists had never or rarely recommended INCs. Since the symptom-based treatment patterns of pharmacists were not surveyed directly and neither was the AR severity of the patients, it is not possible to make a certain judgment. This may reflect a milder degree of symptoms in those patients seeking treatment without a physician’s prescription or there is some deviation from the ARIA guidelines among pharmacists. Further studies on the management of AR symptoms in a community pharmacy from a Northern Cyprus perspective are warranted.

Our intervention study focused on AR education including avoidance of triggers, counselling on medication and detailed training on the technique of administration of nasal spray. Our results suggested pharmacist intervention can improve patient’s outcomes.

Nasal congestion, identified as the most bothersome symptom of AR (as well as reported by healthcare providers and patients in first phase of this study), was significantly improved in the interventional group. Since INCs are considered the most effective medications for controlling symptoms such as nasal congestion, the significant improvement in the interventional group may further reflect the importance of educating patients on techniques of administering nasal sprays and addressing proper use, treatment expectations, patient’s misperceptions about INCs and possible side effects which occur less often with a proper administration technique. All these aspects in patient education could be attributed to the enhancement seen in the interventional group in terms of less nasal congestions [31].

Other published studies have revealed a range of successful methods by means of pharmacist’s interventions in AR management and showed similar results to our study. An Australian study [32] compared two interventions facilitated by pharmacists. In the first group, participants nominated personally their relevant goals and strategies relating to their rhinitis, and in the second, participants had their goals and strategies set by the pharmacist. Although both groups demonstrated significant improvements in symptom severity and quality of life scores, the second group’s severity scores improved more. In a study in Prague, Todorova et al. (2017) explored the impact of pharmaceutical care on improving the QoL in patients with AR [17]. Pharmaceutical care and patient counseling provided in compliance with ARIA guidelines by the pharmacist can successfully enhance the adequate self-management and improve the QoL in patients with intermittent and light persistent AR.

In a UK study, Hammersley et al. (2014) conducted a randomized controlled trial of an educational intervention with general practice staff. Their primary outcome measure was the change in the validated Rhinoconjunctivitis Quality of Life Questionnaire with Standardized Activities (RQLQ(S)) score between baseline and 6 weeks post intervention. The secondary outcome measures of interest include health-care professionals’ knowledge and confidence in managing seasonal allergic rhinitis, the number of seasonal allergic rhinitis-related consultations, relevant treatments prescribed and symptom scores [33]. According to this study, clinically significant improvement in disease-specific quality of life or symptom score in adolescents with seasonal allergic rhinitis was not observed. Furthermore, many intervention programs feature teaching of nasal spray technique as the sole or predominant intervention. An Italian study [34] investigated different types of patient education in the treatment of allergic rhinitis and its effects on nasal and bronchial symptoms. In this study, 101 patients were randomized into three groups. The first group includes drug therapy alone, the second group drug therapy plus training on the use of nasal spray, and a third group drug therapy, nasal spray training and a lesson on rhinitis and asthma. The rate of dropout was highest in the untrained patients. Although no difference in nasal symptoms was seen among the three groups, the third group had significantly fewer asthma symptoms. Therefore, pharmacist-led educational training seems to be important in the management of AR symptoms and QoL of AR patients.

### Limitations of the Study

This study has some limitations that could decrease the generalizability of the results. First, the purposive sampling method was used to recruit ENT specialists which may be subject to bias. However, there are 31 ENT specialists registered with Cyprus Turkish Medical Association and only 19 of those have been carrying out their occupation. Secondly, since the intervention was applied by a limited number of pharmacists, this study may not be sufficient to extrapolate the data to a national context. Despite these limitations, this is the first study to provide an insight into the potential role a pharmacist can play in providing care to AR patients, and thus may be helpful to professional organizations and health authorities interested in optimizing care for such patient groups and those interested in expanding the roles of pharmacists.

## 5. Conclusions

Allergic rhinitis and asthma are common co-morbidities requiring an early intervention by a pharmacist by referring patients to an allergist or properly educating them and following up those diagnosed and prescribed AR medicine. Awareness of the ARIA guidelines among ENT specialists and community pharmacists is low, and treatment has not always been in accordance with the ARIA guidelines. The effect of an educational intervention of the pharmacist provided in compliance with ARIA guidelines indicates that pharmacists can successfully enhance the symptom management and improve the QoL in patients with AR. This study highlights the need for allergy education programs targeted at ENT specialists and community pharmacists to provide better AR management.

## Figures and Tables

**Table 1:** Disease characteristics.

Characteristic	Value
Key AR symptoms for physician diagnosis, *n* (%)	
Nasal congestion	7 (58.3)
Rhinorrhea	3 (25)
Sneezing	1 (8.3)
Nasal itching	2 (16.7)
Ocular symptoms	1 (8.3)
Cough	1 (8.3)
Most bothersome symptom motivating patients to visit the physician, *n* (%)	
Nasal congestion	133 (96.4)
Sneezing	116 (84.1)
Nasal itching	112 (81.2)
Rhinorrhea	121 (87.7)
Cough	118 (85.5)
Ocular symptoms	110 (79.7)
Chief symptoms that made pharmacist suspect AR, *n* (%)	
Nasal congestion	29 (41.4)
Rhinorrhea	30 (42.9)
Sneezing	21 (30)
Nasal itching	14 (20)
Cough	9 (12.9)
Ocular symptoms	10 (14.3)
Causes of symptoms according to patients, *n* (%)	
Animals	9 (6.5)
Plants	44 (31.9)
Seasonal changes	63 (45.7)
Chemicals	21(15.2)
Dust	65 (41.7)
Others	15 (10.9)
Impact on patients’ QoL, *n* (%)	
Impairment of daily activities	24 (17.4)
Sleep disturbances	23 (16.7)
Impairment of work or school	12 (8.7)
Troublesome symptoms	79 (57.2)
None	0 (0)

AR, allergic rhinitis; QoL, quality of life; ENT specialist, ear, nose and throat specialist.

**Table 2:** Medication scores regarding the frequency of recommendation.

	Pharmacists		ENT Specialists	
Medication	Median (IQR)	*N* (%)	Median (IQR)	*N* (%)
Oral antihistamine	3.50 (1)	35 (50)	3 (0)	8 (66.7)
Intranasal antihistamine	2 (2)	26 (37.1)	1 (1)	5 (41.7)
INCs	1 (2)	29 (41.4)	4 (1)	9 (75)
Oral corticosteroids	0 (0)	55 (78.6)	1 (1)	8 (66.7)
Oral decongestants	2 (2)	25 (35.7)	1 (2)	7 (58.3)
Intranasal decongestants	2 (1)	28 (40)	1.50 (1)	5 (41.7)
Anticholinergics	0 (0)	55 (78.6)	1 (2)	5 (41.7)
LTRAs	0 (0)	56 (80)	0.50 (2)	6 (50)
Nasal saline	3 (1)	22 (31.4)	1(1)	6 (50)

INCs, intranasal corticosteroids; LTRAs, leukotriene receptor antagonists; ENT specialist, ear, nose and throat specialist; IQR, interquartile range; median (0 = never; 1 = rarely; 2 = occasionally; 3 = frequently; 4 = very frequently) and *N* (%).

**Table 3:** Impact of pharmacist-led intervention on patients’ symptoms severity according to VAS scores.

Symptom	Group	Baseline (V1) Median Score (IQR)	Final Visit (V2) Median Score (IQR)	Within Group Change Scores *p*-Value ^#^	Between Group Change scores *p*-Value ^^^
Nasal secretions	G1	8 (4)	0 (0)	<0.05	0.23
	G2	8 (4)	0 (0)		
Nasal congestions	G1	9 (2)	1 (0)	<0.05	<0.05
	G2	9 (2)	0 (0)		
Nasal Itching	G1	5 (3)	0 (0)	<0.05	0.65
	G2	5.50 (4.8)	0 (0)		
Sneezing	G1	7 (3)	0 (0)	<0.05	0.35
	G2	8 (3)	0 (0)		
Cough	G1	3 (3)	0 (0)	<0.05	0.35
	G2	4.5 (2.8)	0 (0)		
Eye related	G1	3 (1)	0 (0)	<0.05	0.97
	G2	3 (2.8)	0 (0)		

^#^ Wilcoxon signed ranks test; ^^^ Mann–Whitney U test; G1, control group; G2, intervention group; VAS, visual analogue scale; IQR, interquartile range.

**Table 4:** Impact of pharmacist-led intervention on patients’ QoL according to Mini RQLQ subscale scores.

Symptoms	Group	Baseline (V1) Median Score (IQR)	Final Visit (V2) Median Score (IQR)	Within Group Change Scores *p*-Value ^#^	Between Group Change Scores *p*-Value ^^^
Overall	G1	54 (13)	7 (11)	<0.05	<0.05
	G2	54.50 (13.75)	0 (3)		
Activity limitations	G1	16 (5)	3 (4)	<0.05	<0.05
	G2	16 (3)	0 (0)		
Practical problems	G1	8 (2)	1 (2)	<0.05	<0.05
	G2	8 (3)	0 (0.75)		
Nose symptoms	G1	13 (2)	1 (2)	<0.05	<0.05
	G2	13.5 (2.75)	0 (0.75)		
Eye symptoms	G1	3 (3)	0 (0)	<0.05	0.52
	G2	3 (5.25)	0 (0)		
Other symptoms	G1	13 (3)	2 (3)	<0.05	<0.05
	G2	13 (3.75)	0 (0.75)		

^#^ Wilcoxon signed ranks test; ^^^ Mann–Whitney U test; G1, control group; G2, intervention group; Mini RQLQ, mini rhinoconjunctivitis quality of life (QoL) questionnaire; IQR, interquartile range.
